# Unraveling the patterns and pathways of local recurrence of nasopharyngeal carcinoma: evidence for individualized clinical target volume delineation

**DOI:** 10.1186/s13014-023-02199-3

**Published:** 2023-03-21

**Authors:** Xiaomin Ou, Wenbin Yan, Yangle Huang, Xiayun He, Hongmei Ying, Xueguan Lu, Hui Zhu, Bin Wu, Jiazhou Wang, Chaosu Hu

**Affiliations:** 1 Department of Radiation Oncology, Fudan University Shanghai Cancer Center, 270 Dong’an Road, Shanghai, 200032 China; 2 Department of Oncology, Shanghai Medical College, Fudan University, 270 Dong’an Road, Shanghai, 200032 China; 3 Shanghai Clinical Research Center for Radiation Oncology, 270 Dong’an Road, Shanghai, 200032 China; 4 Shanghai Key Laboratory of Radiation Oncology, 270 Dong’an Road, Shanghai, 200032 China; 5 Department of Radiology, Fudan University Shanghai Cancer Center, 270 Dong’an Road, Shanghai, 200032 China

**Keywords:** Nasopharyngeal carcinoma, Intensity-modulated radiation therapy, Clinical target volume, Local recurrence

## Abstract

**Backgrounds:**

Despite publication of international guidelines, there are notable controversial points of clinical target volume (CTV) delineation in nasopharyngeal carcinoma (NPC). Recently, scholars proposed a novel way of delineation of CTV in NPC—individualization of CTV delineation based on T classification and spread patterns, which yielded excellent long-term local control with limited late toxicities. The aim of this study was to clarify the anatomic patterns and pathways of local recurrence of NPC and provide a clinical reference for the delineation of CTV.

**Methods:**

A total of 869 patients with non-metastatic NPC were treated with intensity-modulated radiation therapy (IMRT) at our institution between 2009 and 2010. Among the 57 cases of local/locoregional recurrence, 52 cases with traceable radiotherapy plans and magnetic resonance imaging at the time of the first diagnosis of recurrence were included. Anatomical structures and gross tumor volume of local recurrence were contoured. The incidence of relapse of each anatomic structure, route of local recurrence, and their correlation were analyzed.

**Results:**

Locally advanced disease had a significantly increased risk of recurrence in the posterior nasal cavity and a trend towards higher risk of recurrence in the clivus, lateral pterygoid muscle, and hypoglossal canal. Based on the incidence of local recurrence, we constructed a high-risk map for the early and locally advanced stages. Local recurrences were classified into five routes, where anterior extension accounted for the majority (30.8%), and caudal tumor extension pathway had the lowest incidence (5.8%). There was a significant correlation between the local recurrences of neural foramina and neighboring anatomical structures along each pathway. All cases relapsed at unilateral cavernous sinus, most at the same side of primary tumor. Based on our findings, we proposed some suggestions on delineations of CTV, based on T classification and local extension pattern.

**Conclusions:**

Local recurrence of NPC varied according to T classification, followed a stepwise pattern, spread via neural foramina, and recurred at ipsilateral cavernous sinus. This provides meaningful clinical evidence for delineation of CTV, especially individualized delineation.

**Supplementary Information:**

The online version contains supplementary material available at 10.1186/s13014-023-02199-3.

## Introduction

Radiotherapy is the primary treatment modality for nasopharyngeal carcinoma (NPC). Given the neighboring relationship between the primary tumor and critical normal organs, target delineation in NPC remains a challenge for radiation oncologists. Even in the era of intensity-modulated radiation therapy (IMRT), inadequate coverage of the target volume or inadequate dosimetry remains one of the most important reasons for local failure [1, 2].

The fundamental step is accurate delineation of the gross tumor volume (GTV). Given the application of multimodal radiology techniques, the contouring of GTV has become increasingly consistent. The high-risk clinical target volume (CTV) is generated by the expansion of 8–10 mm of the GTV, with anatomic edition according to different intra-institutional practice, clinical protocols, or regional guidelines [3–8]. Adequate delineation of high-risk CTV is important for local control. However, there are still notable differences in the delineation of the CTV between various institutions [9]. The recent publication of international guidelines for delineation of the CTV for NPC [10] has provided a practical reference for radiation oncologists; however, most recommendations were the consensus of experts, which were based on the risk of involvement of primary tumor and traditional bony landmarks of 2-dimensional (2D) radiotherapy. According to the guidelines, there were a few controversial points of delineation, which had reached a low degree of agreement and lacked clinical validation.

There are a few studies on the local extension patterns of primary tumors in NPC [11, 12]. However, to our knowledge, there is a paucity of research focusing on concrete analysis of local recurrences of NPC in the era of IMRT. Most studies classified local relapses as in-field, marginal, or out-of-field failures [13–15], yet did not assess the anatomic distributions of local recurrence. We believe that a comprehensive analysis of patterns of local recurrence helps to validate the delineation of CTV and thus provide hints for future modifications. Hence, we collected local recurrence cases in a large consecutive cohort of patients treated by IMRT with long-term follow-up, aiming to perform an in-depth analysis of the local recurrence patterns of NPC.

## Methods

### Patients

From January 2009 to December 2010, 869 patients with primary non-metastatic, pathologically confirmed NPC were treated with IMRT at Fudan University Shanghai Cancer Center. All patients were re-staged using the 8th edition of the American Joint Committee on Cancer (AJCC) [16]. The details of pretreatment evaluation, treatment, and long-term survival were described in a previous publication [17]. In brief, the 5-year local recurrence-free survival was 89.7% with a median follow-up of 54.3 months [17]. In this study, only patients with solitary local recurrence as well as local and regional recurrence (57 cases) were included. All cases with local recurrence in our study were confirmed by re-biopsy. The primary IMRT plans were recovered. Magnetic resonance imaging (MRI) at the time of the first diagnosis of local/locoregional recurrence was traced and imported into the MIM system (7.1.2; MIM Software, Beachwood, OH). This MRI was fused and aligned with the primary computed tomography (CT) of the IMRT plans. The original IMRT plan or MRI of local recurrence was unavailable in five patients because of failure of recovery of IMRT plans and MRI and diagnosis of local relapse in other hospitals. In total, 52 patients with traceable primary radiotherapy plans and MRI for local recurrence were included in this study. A flow diagram of the enrolled patients is shown in Additional file 1: Fig. A.1. The study protocol was approved by the Institutional Review Board (IRB) of Fudan University Shanghai Cancer Center (2208258-14) according to the principles of the Declaration of Helsinki. The informed consent was waived by the IRB due to the retrospective nature, minimal risk to participants and secondary analysis of previous project. The clinical characteristics of the 52 patients are shown in Additional file 1: Table A.1. The median disease-free interval was 35.3 (range 8.9–60.0) months.

### Radiotherapy techniques

Patients were immobilized in the supine position with a thermoplastic mask. CT was performed after immobilization, obtaining 3-mm slices from the calvarium to the hyoid bone and 5-mm slices from the hyoid bone to 2 cm below the sternoclavicular joint. According to the definitions of the ICRU50 and ICRU62 (International Commission on Radiation Units and Measurements), the target volumes were outlined on each layer of the CT images on an IMRT workstation (Pinnacle 3; Philips Healthcare, Best, Netherlands). GTVp included primary tumors and metastatic lymph nodes. The high-risk CTV should cover at least the GTVp with an 8-mm expansion, the entire nasopharynx, parapharyngeal space, at least the anterior two-third of the clivus, the base of the skull, pterygoid fossa, pterygopalatine fossa, inferior sphenoid sinus, and posterior edge of the nasal cavity and maxillary sinuses (at least 5 mm of the posterior part). If the tumor is T3–T4, the entire sphenoid sinus, entire clivus, and bilateral cavernous sinus should be included. The details are outlined in Table 1. The delineation of high-risk CTV of cervical nodes was depicted in our previous publication [18].

**Table 1:** Delineation of clinical target volume of nasopharyngeal carcinoma according to various institutions’ practice, international guidelines, and clinical protocols

	NRG HN001 protocol	Our Institutional protocol	International guidelines	Suggestions based on current study
	GTVp + 8 mm + whole NP	GTVp + 8 mm + whole NP	GTVp + 10 mm + whole NP	GTVp + 8 mm + whole NP
T1-2				
Lateral	Bilateral parapharyngeal space Bilateral pterygoid fossa	Bilateral parapharyngeal space Bilateral lateral pterygoid plates	Bilateral parapharyngeal space Cover pterygoid muscle by 5 + 5 mm expansion from GTVp only, unless there is gross muscular invasion; Cover the whole muscle in case of invasion of the deep fascia/ epimysium of the pterygoid muscle	Bilateral parapharyngeal space Bilateral lateral pterygoid plates (*including medial pterygoid muscle*)
Anterior	Posterior 1/4 of nasal cavity Posterior 1/4 of maxillary sinus	At least 5 mm from choana, posterior wall of maxillary sinus If nasal cavity is involved, then posterior part of ethmoid sinus should be included Pterygoid process PP fossa	At least 5 mm from choana, posterior wall of maxillary sinus Include vomer and the surrounding ethmoid sinus PP fossa	At least 5 mm from choana, posterior wall of maxillary sinus *Include vomer and the surrounding ethmoid sinus* PP fossa
Superior	Skull base (including foramen ovale and rotundum bilaterally) Inferior sphenoid sinus	Skull base including foramen ovale and rotundum, lacerum, petrous apex bilaterally Inferior sphenoid sinus	Skull base including foramen ovale and rotundum, lacerum, petrous apex bilaterally Inferior sphenoid sinus (1/2)	Skull base including foramen ovale and rotundum, lacerum, petrous apex bilaterally Inferior sphenoid sinus (1/2)
Posterior	Anterior 1/3 clivus	Prevertebral muscle Anterior 2/3 clivus	Anterior 1/3 clivus Spare jugular foramen and hypoglossal canal in the absence of extensive posterior-lateral infiltration of primary tumor or high jugular lymphadenopathy	Prevertebral muscle Anterior 1/3 clivus *Spare jugular foramen and hypoglossal canal in the absence of extensive posterior-lateral infiltration of primary tumor or high jugular lymphadenopathy*
Inferior	Not stated	The caudal border of palatine uvula or the midpoint of C2 If oropharynx involved, the downward at least 10 mm	Not stated^a^	The caudal border of palatine uvula or the midpoint of C2 If oropharynx involved, the downward at least 10 mm
T3–4				
Lateral	Bilateral parapharyngeal space Bilateral pterygoid fossa	Bilateral parapharyngeal space Bilateral lateral pterygoid plates If medial pterygoid muscle involved, then lateral pterygoid muscle should be included If lateral pterygoid muscle involved, then infratemporal fossa should be included	Bilateral parapharyngeal space Cover pterygoid muscle by 5 + 5 mm expansion from GTVp only, unless there is gross muscular invasion; Cover the whole muscle in case of invasion of the deep fascia/ epimysium of the pterygoid muscle	Bilateral parapharyngeal space Bilateral lateral pterygoid plates (including medial pterygoid muscle) *If medial pterygoid muscle involved, then lateral pterygoid muscle should be included*
Anterior	Posterior 1/4 of nasal cavity Posterior 1/4 of maxillary sinus	At least 5 mm from posterior wall of nasal cavity and maxillary sinus Include vomer and the surrounding ethmoid sinus Pterygoid process PP fossa	At least 5 mm from choana, posterior wall of maxillary sinus Include vomer and the surrounding ethmoid sinus If sphenoid sinus involved, then upper part of posterior ethmoid sinus should be covered PP fossa	At least 5 mm from choana, posterior wall of maxillary sinus *Included posterior part of ethmoid sinus, in case of involvement of posterior nasal cavity or sphenoid sinus* PP fossa *Include inferior orbital fissure in case of involvement of PP fossa*
Superior	Skull base including foramen ovale and rotundum bilaterally Entire sphenoid sinus Cavernous sinus (involved side only)	Skull base including foramen ovale and rotundum, lacerum, petrous apex bilaterally Entire sphenoid sinus If sphenoid sinus involved, then posterior part of ethmoid sinus If posterior ethmoid sinus involved, then anterior ethmoid sinus should be included Bilateral cavernous sinus	Skull base including foramen ovale and rotundum, lacerum, petrous apex bilaterally Entire sphenoid sinus Cavernous sinus (involved side only)	Skull base including foramen ovale and rotundum, lacerum, petrous apex bilaterally Entire sphenoid sinus *Included posterior part of ethmoid sinus, in case of involvement of posterior nasal cavity or sphenoid sinus* If posterior ethmoid sinus involved, then anterior sphenoid sinus should be included *Cavernous sinus (involved side only)*
Posterior	Anterior 1/3 clivus (entire if involved)	Prevertebral muscle Anterior 2/3 clivus (entire if involved)	Anterior 1/3 clivus (entire if involved) Spare jugular foramen and hypoglossal canal in the absence of extensive posterior-lateral infiltration of primary tumor or high jugular lymphadenopathy	Prevertebral muscle Anterior 1/3 clivus (entire if involved) *Include jugular foramen in case of extensive posterior-lateral infiltration of primary tumor or high jugular lymphadenopathy* *If clivus or jugular foramen involved, then hypoglossal canal should be included*
Inferior	Not stated	The caudal border of palatine uvula or the midpoint of C2 If oropharynx involved, the downward at least 10 mm	Not stated^a^	The caudal border of palatine uvula or the midpoint of C2 If oropharynx involved, the downward at least 10 mm

The italic words were our suggestions based on the results of current study

PP fossa, pterygopalatine fossa; NP, Nasopharynx; C1, the first cervical vertebra; C2, the second cervical vertebra

^a^International guideline does not state the caudal border of high-risk CTV. However, guidelines recommended setting the caudal limit of primary tumor CTV (GTVp + 5 mm + whole nasopharynx) to the caudal border of C1

A margin of 3–5 mm around the GTV and CTV should be added to account for patient motion and setup error. A smaller margin (at least 1–2 mm and 2–3 mm) will be used for the GTV and CTV, which is adjacent to a critical neurological structure.

Radiation was delivered using a simultaneous integrated boost technique. The total dose to the primary tumor was 66 Gy in 30 fractions for T1 or T2 disease, and 70.4 Gy in 32 fractions for T3 or T4 lesions. The total dose delivered to the metastatic lymph nodes was 66 Gy in 30–32 fractions. High- and low-risk CTV received 60 Gy and 54 Gy in 30–32 fractions, respectively. Inverse IMRT plans were optimized using the Pinnacle treatment planning system. Normal tissue contouring, constraints and plan evaluation were in accordance with the Radiation Therapy Oncology Group 0225 protocol [5].

### Contouring anatomic structures and GTVr

Primary MRI was fused with primary CT of the IMRT plan using automated rigid alignment with MIM software. An experienced radiation oncologist contoured normal anatomic structures with the guidance of MRI (Additional file 1: Fig. A.2). The anatomical structures were selected based on the risk assessment of primary tumor extensions, according to the literature [11, 12]. The nomenclature of the anatomic structures is listed in Additional file 1: Table A.2.

Then, MRI of local recurrence was co-registered with primary CT sets of the IMRT plan using automated rigid alignment by MIM software. The delineation of the GTV of the recurrent tumor (GTVr) was first performed with the help of fused recurrent MRI, after which minor manual modification was permitted, considering the anatomic changes between primary CT and recurrent MRI (Additional file 1: Fig. A.2).

Two experienced radiology experts (with more than 15 years of practice) reviewed the contouring of the GTVr and anatomic structures. Any disagreement was resolved by discussion and recontouring.

### Analysis of anatomic distribution of local recurrence and pathways

The delineation of the GTVr and anatomic structures were exported from MIM with DICOM format. A python script was used to calculate the overlap volume between anatomic structures and GTVr. Then the overlap data for each patient were exported to R project (version 4.0.5; R Foundation, Vienna, Austria) for statistics analysis. The anatomic structure was marked as local recurrence if a certain percentage of the volume overlapped with the GTVr. To select the appropriate threshold to define local recurrence, we compared the proportion of cases with certain anatomic structures involved under various thresholds of  volume overlapped with GTVr. As shown in Additional file 1: Fig. A.3 and Table A.3, the pattern under the threshold of ≥ 1% was consistent with that of ≥ 2–5%, whereas the threshold of > 0% seemed to be much more sensitive than the other thresholds. To balance the sensitivity and specificity, we selected a threshold of ≥ 1%; that is, if ≥ 1% of the volume of a certain anatomic structure overlapped with the GTVr, then this structure was defined as local recurrence in this case.

Sanford et al. [19] summarized the local extension patterns of primary tumor into five common routes. We adopted this classification as the pathway of local recurrence and classified each case into a certain pathway manually if at least two neighboring anatomic structures along this pathway were involved in local recurrence.

### Dosimetric analysis

The dose coverage of the entire cohort is presented in Additional file 1: Table A.4. The dosimetric parameters of patients with and without local recurrence are compared in Additional file 1: Table A.5. The dose coverage of T1, T2 was generally better than that of T3,4. However, there were no significant differences in V100, V99, V95,^1^ and D98^2^ of planning tumor volume (PTV) of primary tumor (PTV-G) and V95 and V99 of PTV of high-risk CTV between patients with and without local recurrence in this study.

### Statistical analysis

All analyses were performed using R project. The χ^2^ test or Fisher’s exact test was used to compare categorical variables, and the independent Student’s *t*-test was used to compare the means of continuous variables. Spearman or Pearson correlation was used to analyze the relationship between the two variables. In all cases, a two-sided *p* < 0.05 was considered statistically significant.

## Results

### Risk of local recurrence of various anatomic structures

The cumulative incidence of local recurrence at different anatomical structures varied from 1.9 to 76.9% (Additional file 1: Table A.6). T classification is an important factor that influences the risk of local recurrence. Locally advanced disease (T3–T4) had a significantly higher risk of recurrence at the posterior part of the nasal cavity (52.6% vs. 21.2%, *p* = 0.020) and showed a trend towards a higher risk of recurrence at the clivus (42.1% vs. 18.2%, *p* = 0.061), lateral pterygoid muscle (21.1% vs. 3.0%, *p* = 0.054), and hypoglossal canal (21.1% vs. 3.0%, *p* = 0.054).

According to the cumulative incidence or local recurrence, we classified anatomical structures into three risk grades: high risk (≥ 30%), median risk (≥ 15–30%), and low risk (< 15%), as shown in Table 2. In locally advanced cases (T3–T4), these anatomical structures had a median risk of local recurrence and were recommended being covered by CTV, including the posterior ethmoid sinus (21.1%), lateral pterygoid muscle (21.1%), hypoglossal canal (21.1%), inferior orbital fissure (15.8%), and cavernous sinus (15.8%).

**Table 2:** Risk classification of local recurrence at various anatomic sites and recommended delineation of clinical target volume

T1-2(N = 33)				T3-4(N = 19)			
Anatomic sites	No	Percentage	Risk of recurrence	Anatomic sites	No	Percentage	Risk of recurrence
Levator veli palatine muscle	26	78.8%	High	Levator veli palatine muscle	14	73.7%	High
Nasopharynx	25	75.8%		Nasopharynx	14	73.7%	
Prevertebral muscle	24	72.7%		Prevertebral muscle	11	57.9%	
Tensor veli palatine muscle	20	60.6%		Tensor veli palatine muscle	11	57.9%	
Retropharyngeal lymph node	20	60.6%		Foramen lacerum	11	57.9%	
Foramen lacerum	17	51.5%		Posterior Nasal cavity	10	52.6%	
Bottom of sphenoid sinus	17	51.5%		Great wing of sphenoid bone	9	47.4%	
Pterygoid process	14	42.4%		Bottom of sphenoid sinus	9	47.4%	
Great wing of sphenoid bone	11	33.3%		Pterygoid process	8	42.1%	
Parapharyngeal space	11	33.3%		Petrous apex	8	42.1%	
Sphenoid sinus	10	30.3%		Clivus	8	42.1%	
Petrous apex	8	24.2%	Median	Retropharyngeal lymph node	7	36.8%	
Posterior Nasal cavity	7	21.2%		Pterygopalatine fossa	7	36.8%	
Jugular Foramen	7	21.2%		Parapharyngeal Space	6	31.6%	
Clivus	6	18.2%		Sphenoid sinus	6	31.6%	
Pterygopalatine fossa	6	18.2%		Jugular Foramen	5	26.3%	Median
Medial pterygoid muscle	6	18.2%		Posterior ethmoid sinus^a^	4	21.1%	
Foramen ovale	6	18.2%		Hypoglossal canal^a^	4	21.1%	
Foramen rotundum	4	12.1%	Low	Lateral pterygoid muscle^a^	4	21.1%	
Posterior ethmoid sinus	3	9.1%		Foramen rotundum^a^	3	15.8%	
Inferior orbital fissure	3	9.1%		Inferior orbital fissure^a^	3	15.8%	
Anterior ethmoid sinus	3	9.1%		Cavernous sinus^a^	3	15.8%	
Oropharynx	2	6.1%		Medial pterygoid muscle^b^	2	10.5%	Low
Anterior Nasal cavity	2	6.1%		Orbit	2	10.5%	
Frontal sinus	2	6.1%		Foramen ovale^b^	1	5.3%	
Hypoglossal canal	1	3.0%		Anterior ethmoid sinus	1	5.3%	
Lateral pterygoid muscle	1	3.0%		Oropharynx	1	5.3%	
Cavernous sinus	1	3.0%		Anterior Nasal cavity	1	5.3%	
Orbit	1	3.0%		Maxillary sinus	1	5.3%	
Maxillary sinus	1	3.0%		Cervical vertebrae	1	5.3%	
Cervical vertebrae	0	0.0%		Frontal sinus	0	0.0%	
Infratemporal fossa	0	0.0%		Infratemporal fossa	0	0.0%	
Hypopharynx	0	0.0%		Hypopharynx	0	0.0%	

The anatomic sties with median or high risk should be included in the contouring of CTV

^a^The possibility of invasion of these structures was higher in cases with T3-4 (≥ 15%) than cases with T1-2 (< 15%). These anatomic structures were recommended to be included in CTV in locally advanced cases (T3-4)

^b^Although the possibility of invasion of these structures was low in T3-4 cases (< 15%), they were recommended to be included due to median risk of involvement in early-stage diseases (T1-2)

**Fig. 1:**
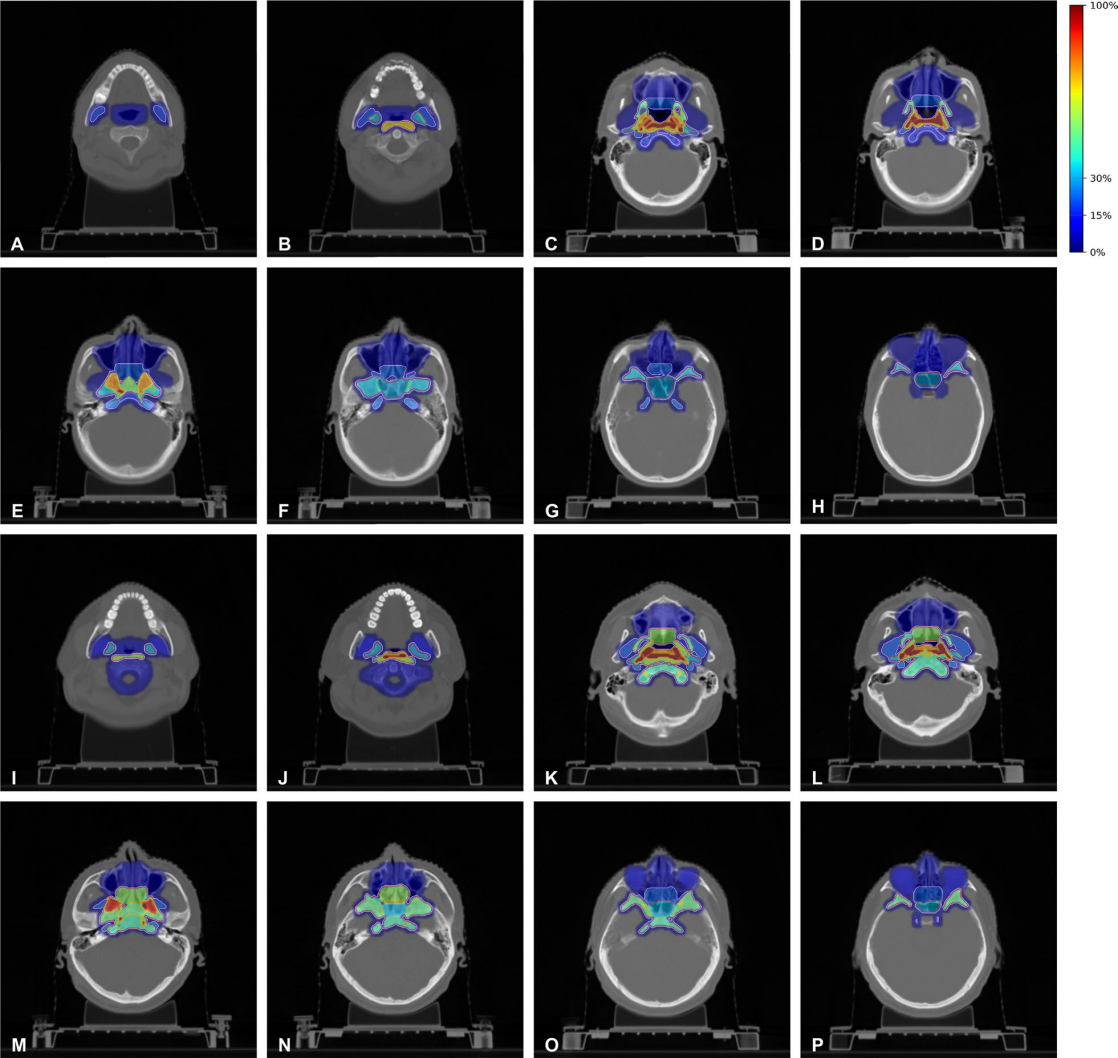
Recommended clinical target volume according to the percentage of local recurrence in cases of early and locally advanced stage of nasopharyngeal carcinoma. **A**–**H** Demonstrated the risk of local recurrence of each anatomic structure and recommended delineation of clinical target volume (CTV) in T1–T2 case with nasopharyngeal carcinoma, at the level of cranial border of C2, midpoint of C1, hard palate, midpoint of maxillary sinus, foramen lacerum, great wing of sphenoid bone, inferior orbital fissure and midpoint of orbit. **I**–**P** Demonstrated the risk of local recurrence of each anatomic structure and recommended delineation of clinical target volume (CTV) in T3–T4 case with nasopharyngeal carcinoma, at the level of cranial border of C2, midpoint of C1, hard palate, midpoint of maxillary sinus, foramen lacerum, great wing of sphenoid bone, inferior orbital fissure and midpoint of orbit. The color bar showed the risk of local recurrence. The pink and red line showed the iso-risk line of 15% and 30% of local recurrence. In our study, the iso-risk line of 15% was selected as the recommended contouring of CTV. It was noteworthy that lateral pterygoid muscle, posterior ethmoid sinus, inferior orbital fissure and cavernous sinus were not included in the delineation of CTV in early-stage disease, but were encompassed by CTV in locally advanced disease

However, in early-stage disease (T1–T2), these structures may not be routinely included in the delineation of the CTV, given the low risk of local recurrence: posterior ethmoid sinus (9.1%), inferior orbital fissure (9.1%), lateral pterygoid muscle (3.0%), hypoglossal canal (3.0%), and cavernous sinus (3.0%). Based on the differences in local recurrence risk, our team generated high-risk maps of local recurrence for early-stage and locally advanced cases using a python script (Fig. 1).

### Pathways of local recurrence

**Fig. 2:**
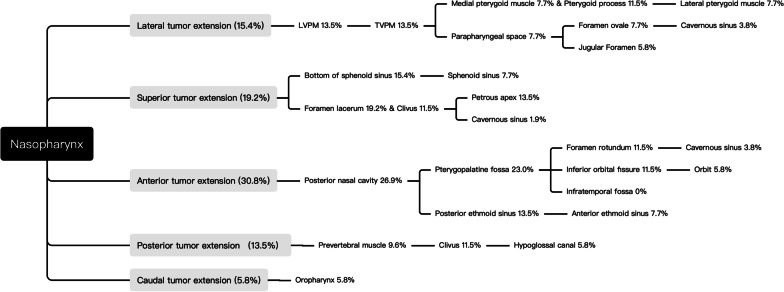
The stepwise pattern and pathways of local recurrence of nasopharyngeal carcinoma. The incidence of recurrence of each anatomic site was listed in the schema. It was noteworthy that most of the recurrence of nasopharyngeal carcinoma followed a stepwise, orderly pattern, with decreased incidence of relapse as the distance from nasopharynx increased. Of note, the cases of in-situ recurrence were excluded from analysis. LVPM, Levator veli palatine muscle; TVPM, Tensor veli palatine muscle

We classified each case of local relapse into five routes (Table 3; Fig. 2) according to the suggestion by Sanford et al. [19]. Cases with local recurrence at the nasopharynx and retropharyngeal lymph nodes were classified as in situ relapse, which was not included in the pathway analysis. Except in situ relapse, all the remaining cases of recurrence fell into the five routes mentioned above.

**Table 3:** The pathways of local recurrences of nasopharyngeal carcinoma

Pathways	Details of each pathway	Count	Percentage (%)
Lateral tumor extension	Nasopharynx-LVPM-Pharyngobasilar fascia-TVPM-Medial pterygoid muscle & Parapharyngeal space-Foramen ovale-Cavernous sinus; or Nasopharynx-LVPM- Pharyngobasilar fascia-Jugular foramen	8	15.4
Superior tumor extension	Nasopharynx-Bottom of sphenoid sinus- Sphenoid sinus; or Nasopharynx-Clivus & Foramen lacerum -Cavernous sinus	10	19.2
Anterior tumor extension	Nasopharynx-Posterior Nasal Cavity-Pterygopalatine fossa-Foramen rotundum & Inferior orbital fissure- Cavernous sinus & Orbit; or Nasopharynx-Posterior Nasal Cavity-Posterior ethmoid sinus-Anterior ethmoid sinus	16	30.8
Posterior tumor extension	Nasopharynx-Prevertebral muscle- Clivus & Hypoglossal canal	7	13.5
Caudal tumor extension	Nasopharynx-oropharynx	3	5.8

LVPM, Levator veli palatine muscle; TVPM, Tensor veli palatine muscle

The most common route of local recurrence was anterior tumor extension (30.8%), in which local relapse mainly occurred through the pterygopalatine fossa. The caudal tumor extension pathway had the lowest incidence of local relapse (5.8%).

Within each pathway, there was a significant correlation between the neural foramina and other anatomic structures (Spearman correlation test, *p* < 0.05, Table 4), and the incidence of recurrence gradually decreased as the distance from the nasopharynx increased (Fig. 2). This indicated that recurrent tumors are likely to spread through neural foramina in a stepwise fashion.

**Table 4:** The correlation between key anatomic structure and other anatomic structures along the same pathway

Spearman correlation test of anatomic sites within the same path, significance level (*p* value)							
Pathway	Key structure	Neighboring structures					
Anterior extension		Posterior Nasal cavity	Pterygoid process	Pterygopalatine fossa	Foramen rotundum	Cavernous sinus	Orbit
	Inferior orbital fissure	0.000*	0.031*	0.000*	0.000*	0.012*	0.232
		Posterior Nasal cavity	Pterygoid process	Pterygopalatine fossa	Inferior orbital fissure	Cavernous sinus	Orbit
	Foramen rotundum	0.001*	0.012*	0.000*	0.000*	0.026*	0.308
		Posterior Nasal cavity	Pterygoid process	Foramen rotundum	Inferior orbital fissure	Cavernous sinus	Orbit
	Pterygopalatine fossa	0.000*	0.000*	0.000*	0.000*	0.238	0.737
		Posterior Nasal cavity	Sphenoid sinus	Anterior ethmoid sinus			
	Posterior ethmoid sinus	0.001*	0.012*	0.000*			
Superior extension		Bottom of sphenoid sinus	Clivus	Petrous apex	Cavernous sinus		
	Foramen lacerum	0.001*	0.000*	0.008*	0.875		
Lateral extension		Levator veli palatine muscle	Tensor veli palatine muscle	Parapharyngeal Space	Medial pterygoid muscle	Cavernous sinus	
	Foramen ovale	0.562	0.136	0.547	0.031*	0.026*	
		Levator veli palatine muscle	Tensor veli palatine muscle	Retropharyngeal lymph node	Parapharyngeal Space		
	Jugular Foramen	0.031*	0.058	0.001*	0.000*		
		Medial pterygoid muscle	Pterygoid process				
	Lateral pterygoid muscle	0.003*	0.075				
Posterior extension		Prevertebral muscle	Clivus	Jugular Foramen			
	Hypoglossal canal	0.105	0.000*	0.000*			

*p < 0.05, indicated significant correlation

In addition, we evaluated the correlation between neighboring structures and anatomic structures with notably different risks of relapse among various T classifications (Table 4). For example, invasion of the inferior orbital fissure was associated with recurrence in the posterior nasal cavity, pterygoid process, pterygopalatine fossa, foramen rotundum, and cavernous sinus. Relapse of the hypoglossal canal was associated with the clivus and jugular foramen. In addition, there were significant correlations between relapse in posterior ethmoid sinus and posterior nasal cavity/ sphenoid sinus, relapse in lateral pterygoid muscle and medial pterygoid muscle. This indicated that certain anatomic structure should be included by CTV in case of the involvement of neighboring structures.

### The unilateral recurrence of cavernous sinus

There were four cases with recurrence of cavernous sinus (Table 5). All four cases relapsed at unilateral cavernous sinus. Among them, three cases were primarily diagnosed as locally advanced disease, and recurred at the same side of the primary tumor. One case had primary T1 disease involving bilateral roof of nasopharynx, however, developed extensive recurrence to cavernous sinus.

**Table 5:** Clinical characteristics of patients with recurrence of cavernous sinus

Patient ID	Primary T stage	Primary involvement of cavernous sinus/skull base	Primary involved neural foramina	Recurrent of cavernous sinus	Recurrent involved neural foramina
No.1	T4	Left	Foramen lacerum, Foramen ovale	Left	Foramen ovale
No.2	T4	Left	Foramen rotundum, Pterygopalatine fossa, Foramen ovale, Foramen lacerum	Left	Foramen rotundum, Pterygopalatine fossa
No.3	T1^a^	No	No	Left	Foramen ovale, Foramen rotundum, Foramen lacerum
No.4	T3	Right	Foramen lacerum	Right	Foramen lacerum, Jugular Foramen

^a^This patient had primary bilateral invasion of the roof of nasopharynx

### Suggestions on delineations of clinical target volumes

As mentioned above, we have depicted high risk maps of local recurrence of various T classifications, evaluated the correlations of local recurrence between key anatomic structure and neighboring structures, and clarified the recurrence pattern at cavernous sinus. Based on current findings, we propose some suggestions on delineation of CTV, which are summarized in Table 1.

For T1–T2 disease, we suggest including medial pterygoid muscle in CTV laterally, given the median risk of local relapse (18.2%) at this structure. Our data demonstrated the low risk of recurrence at posterior ethmoid sinus, so we suggest encompassing the vomer and surrounding ethmoid sinus in CTV to ensure coverage of superior border of nasopharynx, per recommendation of international guideline [10]. Our data showed that the risk of local recurrence at hypoglossal canal in T1–T2 cases was low (3.0%), and the risk of recurrence of this structure correlated with that of clivus and jugular foramen. In addition, the risk of recurrence of jugular foramen correlated with that of levator veli palatine muscle, retropharyngeal nodes and parapharyngeal space. Hence, we recommend sparing the jugular foramen and hypoglossal canal unless there is extensive posterior-lateral infiltration of primary tumor or high jugular lymphadenopathy. We recommend sparing cavernous sinus by CTV in early-stage disease, due to the low incidence of relapse in cavernous sinus.

In T3–T4 disease, local recurrence risk of lateral pterygoid muscle was demonstrated to be marginally higher, and this was significantly associated with invasion of medial pterygoid muscle. Hence, we suggest including lateral pterygoid muscle in CTV in case of the involvement of medial pterygoid muscle in locally advanced disease. Similarly, we advocate including posterior part of ethmoid sinus in case of invasion of posterior nasal cavity or sphenoid sinus, and recommend coverage of inferior orbital fissure in case of involvement of pterygopalatine fossa, given the high risk of local relapse of these anatomic structures and their close relationships in locally advanced cases. Considering the unilateral recurrence of cavernous sinus and the median risk of relapse in T3–T4 disease, we recommend coverage of cavernous sinus of the involved side by CTV.

Of note, these were merely suggestions derived from retrospective data of our institutional practice. The further interpretation of these suggestions should be based on full consideration of individual patient’s factors and the experience of individual center.

## Discussion

The current recommendations of delineation of CTV are generally based on the understanding of the natural behavior of tumor invasion, the experience of 2D/3D radiotherapy, as well as the patterns of local recurrence in the era of IMRT. In this study, we collected local recurrence cases in a large consecutive cohort of patients with uniform intra-institutional protocol and a long-term follow-up, which is one of the few attempts to perform an in-depth analysis of the local recurrence patterns of NPC and provided validations for delineation of CTV.

When IMRT was introduced into the treatment of NPC in early 2000s, the delineation of CTV was universal, irrespective of T classification [5, 7]. Gradually, scholars began to modify the delineation according to T classification. For example, cavernous sinus was included merely in cases of T3–T4 disease, as mentioned in the protocol of RTOG 0615 [20]. Similarly, our study demonstrated that the recurrence risk of anatomic sites varied between various T subgroups. Lateral pterygoid muscle and hypoglossal canal, tended to have a higher incidence of relapse in T3–T4 disease than in T1–T2. The risk of recurrence of cavernous sinus was numerically higher in locally advanced disease, albeit did not reach statistical significance (T3–T4 15.8% vs. T1–T2 3.0%).

In addition, our data showed that certain anatomic structure were exposed to a high risk of local recurrence in case of the involvement of neighboring structures. For example, relapse in the jugular foramen correlated with the levator veli palatini muscle, retropharyngeal node, and parapharyngeal space. Recurrence in the hypoglossal canal was associated with the clivus and jugular foramen. Both supported the practice to cover jugular foramen and hypoglossal canal in case of postero-lateral extension of primary tumor and retropharyngeal lymph nodes, which was consistent with the recommendations of international guidelines of delineation of CTV [10]. Likewise, we propose covering the posterior ethmoid sinus in T3–T4 cases, especially in cases of invasion into the posterior nasal cavity and sphenoid sinus. We suggest encompassing the inferior orbital fissure when primary tumor involved the pterygopalatine fossa and foramen rotundum. Of note, the criteria to cover inferior orbital fissure has not been discussed in the international guidelines before [10].

As for cavernous sinus, Liang et al. [11] reported the risk of bilateral invasion of cavernous sinus was low (1.5%). Sanford et al. [19] advocated covering the Meckel’s cave and cavernous sinus ipsilaterally for lateralized tumors. Wu et al. [21] recently characterized the locoregional extension patterns of unilateral NPC and demonstrated that contralateral skull base was not invaded in this case. In accordance with these, our study showed all cases relapsed at unilateral cavernous sinus, most cases at the same side of the primary tumor. The risk of recurrence of cavernous sinus was higher in locally advanced disease. This supported the recommendations of international guidelines—to spare the cavernous sinus in T1–T2 disease and cover the whole ipsilateral cavernous sinus in T3–T4 disease [10].

Furthermore, our study showed that local recurrence followed similar routes with primary invasions. Sanford et al. [19] summarized local invasions routes into five pathways. Our study showed all local recurrence except in situ relapse could be classified into these five pathways as well. Among five routes, anterior tumor extension (30.8%) accounted for the majority, in which local relapse mainly occurred through the pterygopalatine fossa. This may be partially attributed to the extensive communications of pterygopalatine fossa with various cranial nerves and anatomic structures.

Recently, scholars proposed a novel way of delineation of CTV in NPC—individualization of CTV delineation based on stepwise spread patterns. Li et al. [22] and Sanford et al. [19] adopted different individualized delineations based on T classification and spread patterns, both attained excellent long-termed local control and low late toxicities. Xie et al. [23] reported individualized CTV delineation in unilateral NPC yielded excellent long-term local control with limited out-of-field recurrences. In this cohort, unilateral NPC was defined as a nasopharyngeal mass confined to one side of the nasopharynx and did not exceed the midline [23]. The CTVs were determined based on the distance from the gross tumor, and the contralateral para-pharyngeal space and skull base orifices were spared from irradiation [23]. Our study proved that local recurrence differed among various primary T classifications, followed a stepwise pattern, and shared similar routes with primary invasion, which supported the rationale of individualization of CTV delineation based on T classification and spread patterns. Taken together, individualized CTV delineation is a promising treatment de-escalation strategy that maintains local control and meanwhile spares unnecessary irradiation, which deserves further optimization and validation by prospective clinical studies.

## Conclusions

Collectively, our study demonstrated that local recurrence of NPC varied according to T classification, followed a stepwise pattern, shared similar routes with primary invasions, and usually recurred at ipsilateral cavernous sinus. Despite the retrospective and single-institutional analysis, our study provides a meaningful clinical reference for delineation of high-risk CTV in NPC and supports the rationale of individualized delineation based on T classification and spread patterns. Given the limitations of sample size and retrospective nature, our findings should be further evaluated using external data or prospective clinical studies.

## Supplementary Information

**Additional file 1.** Supplementary Tables and Figures.

## Data Availability

The data in our study have been deposited on the Mendeley Data (data.mendeley.com). After publication of the study findings, our data are available from the corresponding author and database administrator on reasonable request.

## Abbreviations

NPC: Nasopharyngeal carcinoma
GTV: Gross tumor volume
CTV: Clinical tumor volume
IMRT: Intensity-modulated radiation therapy
2D: 2-Dimensional
3D: 3-Dimensional
AJCC: American Joint Committee of Cancer
ICRU: International Commission on Radiation Units and Measurements
GTVp: Gross tumor volume of primary tumor
GTVr: Gross tumor volume of local recurrence
PTV: Planning tumor volume
PTV-G: Planning tumor volume of primary tumor
MRI: Magnetic resonance imaging
CT: Computed tomography
IRB: Institutional Review Board

## Footnotes

1 V95: volume of PTV that received at least 95% of the prescription dose.

2 D98: dose received by 98% of PTV. 
