# Supplementary material for: Unraveling the patterns and pathways of local recurrence of nasopharyngeal carcinoma: evidence for individualized clinical target volume delineation
Source: Radiat Oncol. 2023 Mar 21;18:55. doi: 10.1186/s13014-023-02199-3 (PMC10032020; doi:10.1186/s13014-023-02199-3)
Supplement: Supplementary file 1 — Additional file 1. Supplementary Tables and Figures. [file 13014_2023_2199_MOESM1_ESM.pdf]

Supplementary Materials

Supplementary Materials 1
Table A.1. The clinical characteristics of 52 patients with nasopharyngeal carcinoma with local recurrence 2
Table A.2. The list and abbreviations of all the contouring anatomic structures. 3
Table A.3. The comparison of various thresholds of defining local recurrence 4
Table A.4. The dose coverage of the entire cohort (N=869) 5
Table A.5. The dose coverage of those with/without local recurrence 6
Table A.6. The involved structures of local recurrences of nasopharyngeal carcinoma 7
Figure A.1. Flow diagram of the enrolled patients 8
Figure A.2. The delineation of anatomic structures and GTVr 9
Figure A.3. The comparison of the proportions of cases with certain anatomic structure of local recurrence under various thresholds 10

Table A.1. The clinical characteristics of 52 patients with nasopharyngeal carcinoma with local recurrence

Clinical Factor	No.	Percentage (%)
Age (Median, Range, years)	47	30-80
Gender		
Male	39	75.0
Female	13	25.0
T stage		
T1	16	30.8
T2	17	32.7
T3	13	25.0
T4	6	11.5
N stage		
N0	8	15.4
N1	23	44.2
N2	15	28.8
N3	6	11.5
Clinical stage		
I	4	7.7
II	15	28.8
III	22	42.3
IVA	11	21.1
Pathology		
Non-keratinizing carcinoma, undifferentiated	52	100.0
Treatment strategies		
Radiation	9	17.3
Induction chemotherapy + Radiation	7	13.5
Concurrent chemoradiation +/- adjuvant chemotherapy	4	7.7
Induction chemotherapy+ Concurrent chemoradiation	19	36.5
Induction chemotherapy+ Adjuvant chemotherapy	13	25.0
Complete entire treatment of radiation		
Yes	52	100
Disease-free interval (Median, Range, Months)	35.3	8.9-60.0
Disease-free interval ≤ 2 years	15	28.8
Disease-free interval >2 years and ≤3 years	12	23.1
Disease-free interval > 3 years	25	48.1

Table A.2. The list and abbreviations of all the contouring anatomic structures.

Anatomic structures	Abbreviations
**High Risk***	
Nasopharynx	Nasopharynx
Levator veli palatine muscle	LVPM
Tensor veli palatine muscle	TVPM
Parapharyngeal Space	PP Space
Retropharyngeal lymph node	RLN
Posterior 1/3 of Nasal cavity	Nasal cavity P
Bottom of sphenoid sinus	S sinus bottom
Pterygoid process	P process
Prevertebral muscle	PVM
Foramen lacerum	Foramen L
Clivus	Clivus
Petrous apex	P apex
**Medium Risk***	
Foramen ovale	Foramen O
Great wing of sphenoid bone	S wing
Oropharynx	Oropharynx
Medial pterygoid muscle	MPM
Cavernous sinus	Cavernous S
Pterygopalatine fossa	PP fossa
Sphenoid sinus	S sinus
Hypoglossal canal	HG canal
Lateral pterygoid muscle	LPM
Foramen rotundum	Foramen R
Jugular Foramen	Foramen J
Ethmoid sinus (Posterior part)	E sinus P
Ethmoid sinus (Anterior part)	E sinus A
**Low risk***	
Orbit	Orbit
Inferior orbital fissure	IOF
Infratemporal fossa	IFT fossa
Cervical vertebrae	CV
Maxillary sinus	M sinus
Anterior 2/3 of Nasal cavity	Nasal cavity A
Frontal sinus	F sinus
Hypopharynx	Hypopharynx

* The different risk subgroups referred to the risk of primary invasion, according to the literature [11,12].

Table A.3. The comparison of various thresholds of defining local recurrence

Structure / Threshold*	>0% overlapped with GTVr	≥1% overlapped with GTVr	≥2% overlapped with GTVr	≥3% overlapped with GTVr	≥5% overlapped with GTVr
LVPM	42	39	38	36	31
Nasopharynx	43	37	36	34	33
PVM	42	34	31	28	21
TVPM	38	31	23	20	18
RLN	28	27	27	27	27
Foramen L	28	27	27	25	24
S sinus bottom	26	26	26	26	25
P process	28	22	22	18	17
S wing	26	20	18	16	11
Nasal cavity P	32	17	16	14	13
PP Space	25	16	13	8	7
P apex	24	16	16	11	9
S sinus	17	16	14	14	12
Clivus	28	13	12	12	12
PP fossa	14	13	13	13	13
Foramen J	13	12	11	10	8
MPM	13	8	4	3	2
Foramen O	7	7	7	7	5
Foramen R	7	7	7	6	6
E sinus P	8	7	5	5	4
IOF	7	6	5	5	4
HG canal	6	5	4	4	4
LPM	9	5	2	1	0
Cavernous S	4	4	4	4	3
E sinus A	5	4	4	4	4
Oropharynx	7	3	3	2	0
Orbit	3	3	1	0	0
Nasal cavity A	7	3	3	3	2
M sinus	12	2	2	2	1
F sinus	2	2	2	2	1
CV	7	1	1	1	1
IFT fossa	4	0	0	0	0
Hypopharynx	0	0	0	0	0

* the number of cases with certain anatomic structure relapsed under certain threshold among the whole cohort with local recurrence (N=52). For example, 39 cases had local recurrence at LVPM, under the threshold of ≥1% of volume of LVPM overlapped with GTVr.

Note: the details of abbreviations were demonstrated in Table A.2.

Table A.4. The dose coverage of the entire cohort（N=869)

	D98 of PTV-G	V95 of PTV-G	V99 of PTV-G	V100 of PTV-G	V95 of PTV-60	V99 of PTV-60
T1-2 (N=526)	64.9+/- 3.5Gy	99.0+/- 3.7%	94.8+/- 5.4%	91.0+/-6.5%	97.8+/- 4.1%	93.8+/-5.1%
T3-4 (N=343)	65.9+/- 3.4Gy	96.1+/- 10.8%	89.6+/- 12.5%	85.2+/-13.2%	96.4+/- 10.3%	92.5+/-10.6%
p value	0.000	0.000	0.000	0.000	0.026	0.048

Note: Average value +/- standard deviation, *t*-test.

Table A.5. The dose coverage of those with/without local recurrence

	D98 of PTV-G	V95 of PTV-G	V99 of PTV-G	V100 of PTV-G	V95 of PTV-60	V99 of PTV-60
Those without local recurrence (N=812)	65.3+/-3.6 Gy	97.9+/-7.8%	92.7+/-9.4%	88.7+/-10.2%	97.2+/-7.5%	93.2+/-8.0%
Those with local recurrence (N=57)	65.1+/-2.5 Gy	98.3+/-4.0%	92.7+/-8.0%	88.6+/-9.5%	97.7+/-3.3%	94.1+/-4.5%
p value	0.630	0.692	0.915	0.963	0.669	0.408

Note: Average value +/- standard deviation, *t*-test.

Table A.6. The involved structures of local recurrences of nasopharyngeal carcinoma

Anatomic structures	Total number	Percentage	T1-2(N=33)	Percentage	T3-4(N=19)	Percentage	χ^2 test, *p* value
Levator veli palatine muscle	40	76.9%	26	78.8%	14	73.7%	0.739
Nasopharynx	39	75.0%	25	75.8%	14	73.7%	1
Prevertebral muscle	35	67.3%	24	72.7%	11	57.9%	0.272
Tensor veli palatine muscle	31	59.6%	20	60.6%	11	57.9%	0.848
Foramen lacerum	28	53.8%	17	51.5%	11	57.9%	0.657
Retropharyngeal lymph node	27	51.9%	20	60.6%	7	36.8%	0.099
Bottom of sphenoid sinus	26	50.0%	17	51.5%	9	47.4%	0.773
Pterygoid process	22	42.3%	14	42.4%	8	42.1%	0.982
Great wing of sphenoid bone	20	38.5%	11	33.3%	9	47.4%	0.316
Parapharyngeal Space	17	32.7%	11	33.3%	6	31.6%	0.897
Posterior Nasal cavity	17	32.7%	7	21.2%	10	52.6%	0.020*
Petrous apex	16	30.8%	8	24.2%	8	42.1%	0.179
Sphenoid sinus	16	30.8%	10	30.3%	6	31.6%	0.924
Clivus	14	26.9%	6	18.2%	8	42.1%	0.061#
Pterygopalatine fossa	13	25.0%	6	18.2%	7	36.8%	0.187
Jugular Foramen	12	23.1%	7	21.2%	5	26.3%	0.739
Medial pterygoid muscle	8	15.4%	6	18.2%	2	10.5%	0.694
Foramen ovale	7	13.5%	6	18.2%	1	5.3%	0.242
Foramen rotundum	7	13.5%	4	12.1%	3	15.8%	0.697
Posterior ethmoid sinus	7	13.5%	3	9.1%	4	21.1%	0.400
Inferior orbital fissure	6	11.5%	3	9.1%	3	15.8%	0.656
Hypoglossal canal	5	9.6%	1	3.0%	4	21.1%	0.054#
Lateral pterygoid muscle	5	9.6%	1	3.0%	4	21.1%	0.054#
Cavernous sinus	4	7.7%	1	3.0%	3	15.8%	0.132
Anterior ethmoid sinus	4	7.7%	3	9.1%	1	5.3%	1.000
Oropharynx	3	5.8%	2	6.1%	1	5.3%	1.000
Anterior Nasal cavity	3	5.8%	2	6.1%	1	5.3%	1.000
Orbit	3	5.8%	1	3.0%	2	10.5%	0.546
Maxillary sinus	2	3.8%	1	3.0%	1	5.3%	1.000
Frontal sinus	2	3.8%	2	6.1%	0	0.0%	0.527
Cervical vertebrae	1	1.9%	0	0.0%	1	5.3%	0.365
Infratemporal fossa	0	0.0%	0	0.0%	0	0.0%	N.A.
Hypopharynx	0	0.0%	0	0.0%	0	0.0%	N.A.

* Significant difference; # marginal difference. N.A., not available.

Figure A.1. Flow diagram of the enrolled patients

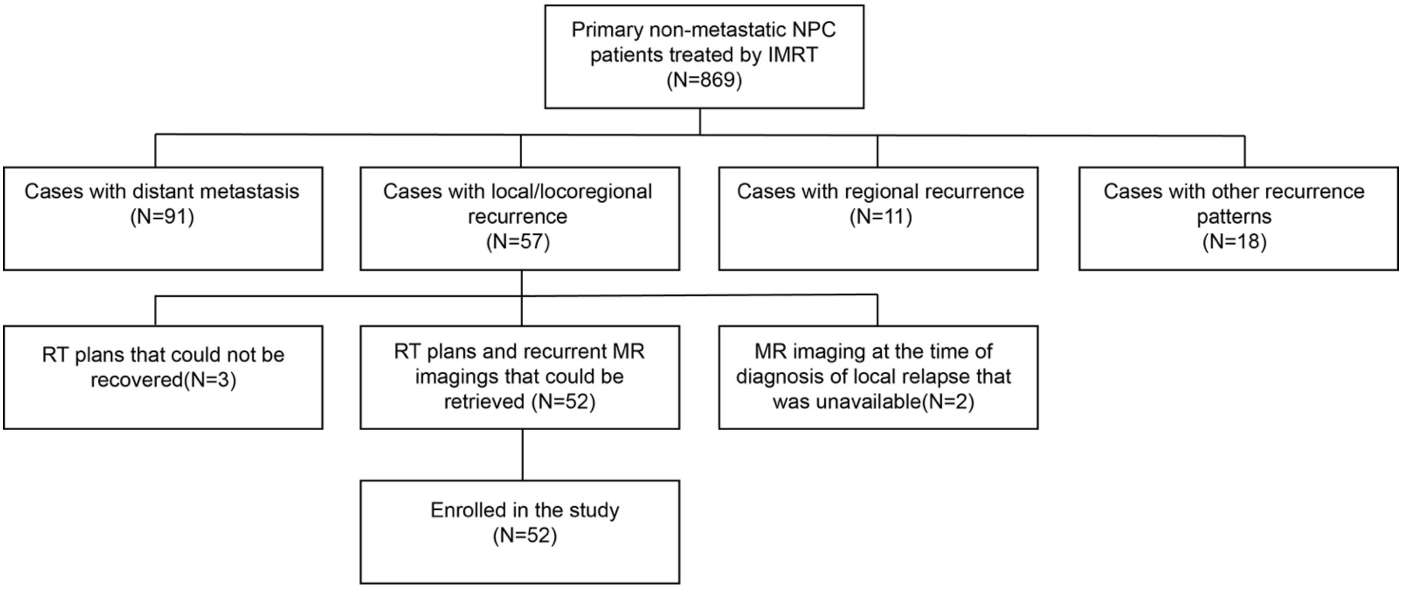

Figure A.2. The delineation of anatomic structures and GTVr

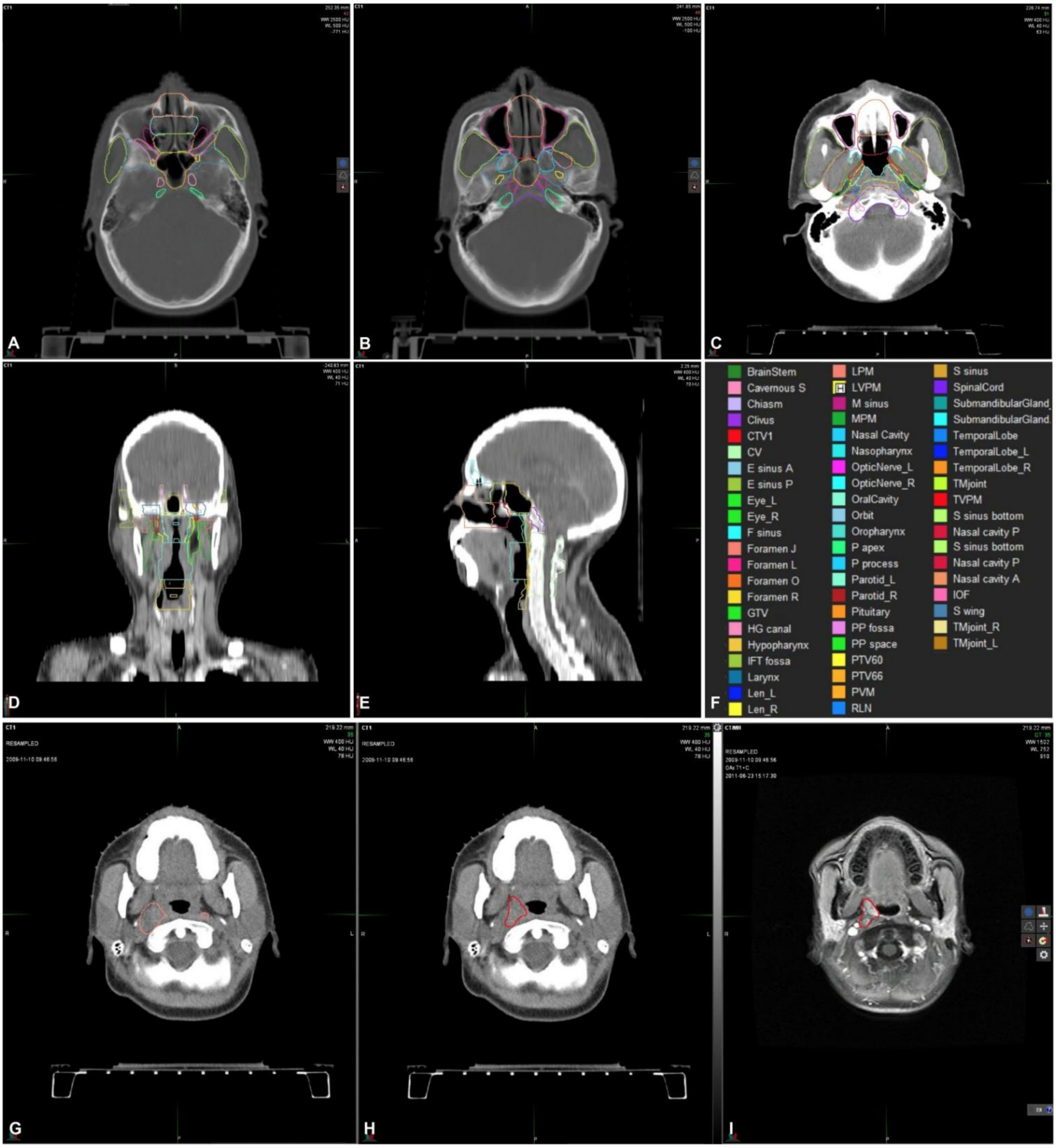

Figure (A), (B), (C) were the axial view of delineation of primary CT sets at various levels. Figure (D), (E) were the coronal, sagittal view, respectively. Figure (F) showed the nomenclature and color of each anatomic structure.

Figure (G), (H) were the primary CT sets at the time of first diagnosis, pink line in (G) was the contouring of GTVp, while red line in (H) was the delineation of GTVr, with the help of the fused MR showing the local recurrence, as demonstrated in Figure (I).

Abbreviations: LVPM, Levator veli palatine muscle; TVPM, Tensor veli palatine muscle; PP Space, Parapharyngeal Space; RLN, Retropharyngeal lymph node; Nasal cavity P, Posterior 1/3 of Nasal cavity; S sinus bottom, Bottom of sphenoid sinus; P process, Pterygoid process; PVM, Prevertebral muscle; Foramen L, Foramen lacerum; P apex, Petrous apex; Foramen O, Foramen ovale; S wing, Great wing of sphenoid bone; MPM, Medial pterygoid muscle; Cavernous S, Cavernous sinus; PP fossa, Pterygopalatine fossa; S sinus, Sphenoid sinus; HG canal, Hypoglossal canal, LPM, Lateral pterygoid muscle; Foramen R, Foramen rotundum; Foramen J, Jugular Foramen; E sinus P, Posterior ethmoid sinus; E sinus A, Anterior ethmoid sinus; IOF, Inferior orbital fissure; IFT fossa, Infratemporal fossa; CV, Cervical vertebrae; Nasal cavity A, Anterior 2/3 of Nasal cavity; M sinus, Maxillary sinus; F sinus, Frontal sinus.

Figure A.3. The comparison of the proportions of cases with certain anatomic structure of local recurrence under various thresholds

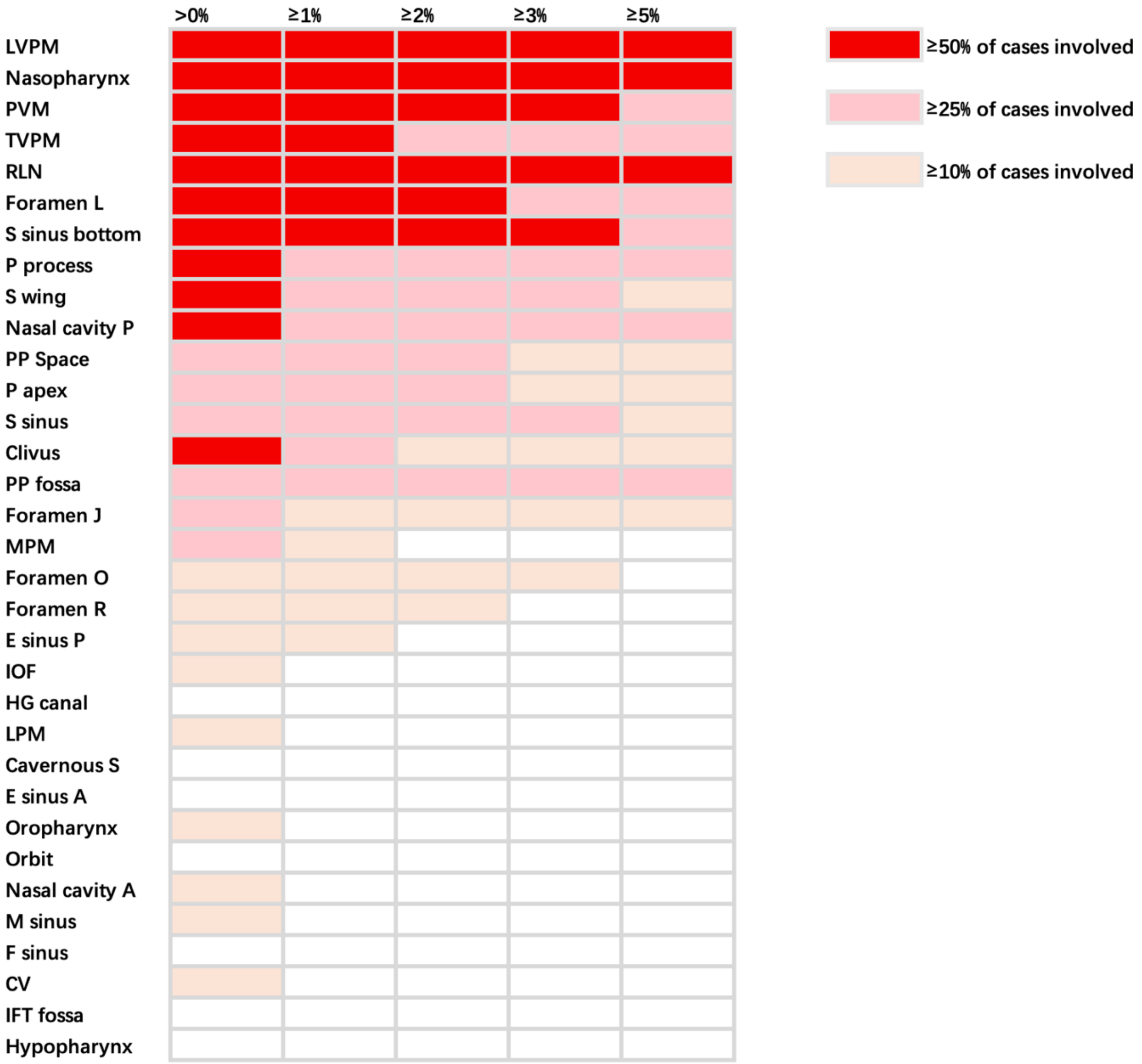

As shown in the figure, the proportion of cases involved under the threshold of ≥1% shared similar pattern with the that of ≥2-5%, while the threshold of > 0% seemed to be much more sensitive than the other thresholds.

Abbreviations: LVPM, Levator veli palatine muscle; TVPM, Tensor veli palatine muscle; PP Space, Parapharyngeal Space; RLN, Retropharyngeal lymph node; Nasal cavity P, Posterior 1/3 of Nasal cavity; S sinus bottom, Bottom of sphenoid sinus; P process, Pterygoid process; PVM, Prevertebral muscle; Foramen L, Foramen lacerum; P apex, Petrous apex; Foramen O, Foramen ovale; S wing, Great wing of sphenoid bone; MPM, Medial pterygoid muscle; Cavernous S, Cavernous sinus; PP fossa, Pterygopalatine fossa; S sinus, Sphenoid sinus; HG canal, Hypoglossal canal, LPM, Lateral pterygoid muscle; Foramen R, Foramen rotundum; Foramen J, Jugular Foramen; E sinus P, Posterior ethmoid sinus; E sinus A, Anterior ethmoid sinus; IOF, Inferior orbital fissure; IFT fossa, Infratemporal fossa; CV, Cervical vertebrae; Nasal cavity A, Anterior 2/3 of Nasal cavity; M sinus, Maxillary sinus; F sinus, Frontal sinus.
